# Treatment of type IIIa endoleak and 7-year follow-up: case report

**DOI:** 10.1590/1677-5449.202400192

**Published:** 2025-07-28

**Authors:** Luiz Ronaldo Godinho Pereira, Keller Soares Ávila, Vinicius Oliveira Godoi, Leonardo Augusto D’Avila Gonçalves, Rafael Fortes, Marcelo Adriano de Assis Hudson, Daniel Mendes Pinto

**Affiliations:** 1 Hospital Márcio Cunha, Ipatinga, MG, Brasil.; 2 Universidade Federal de Juiz de Fora – UFJF, Juiz de Fora, MG, Brasil.; 3 Hospital Felício Rocho, Belo Horizonte, MG, Brasil.

**Keywords:** endoleak, aortic aneurysm, diagnostic imaging

## Abstract

Type III endoleaks are characterized by a problem with the endograft structure, such as fracture of the metallic structure, separation, or rupture. They constitute a rare complication, occurring in 2.1% of patients after treatment of abdominal aortic aneurysm by endovascular repair, with higher incidence in first and second generation endografts, and can occur early (after 30 days) or later. This type III classification is subdivided into IIIa –modular separation of components – and IIIb – mesh fracture or rupture involving the endograft. This case report describes an asymptomatic patient who had previously undergone infrarenal abdominal aortic aneurysm repair and underwent follow-up computed tomography which found a type IIIa endoleak with separation of the main body from the proximal extension. A second endovascular intervention was performed to seal the endoleak and correct the aneurysm.

## INTRODUCTION

Endovascular aneurysm repair (EVAR) is the technique most used to treat abdominal aortic aneurysms (AAA). However, certain complications are possible. The main type of complication is endoleak, which is persistent blood flow within the aneurysm sac after EVAR, accounting for around 33% of complications, although around 50% of cases resolve spontaneously.^1-4^

Endoleaks are classified into five types: type I is subdivided into proximal (Ia) and distal (Ib); type II involves growth of the aneurysm sac due to retrograde blood flow from vessels; type III involves separation of the endograft, whether at the main body or the contralateral extension (IIIa), or a rip or fracture of the endograft (IIIb); type IV is caused by endograft porosity; and type V is aneurysmal growth in the absence of an endoleak (endotension).^1-4^

Treatment of type III endoleaks is obligatory, since there is direct pressure on the aneurysm and a high risk of rupture.^1,5^ This article reports a case in which a fully endovascular technique was used to repair an example of this type of endoleak.

This case report was approved by the Research Ethics Committee at the Hospital Felício Rocho, Belo Horizonte, Brazil, with Ethics Appraisal Submission Certificate 79209524.4.0000.5125 and Consolidated Opinion number: 6.801.488.

## CASE DESCRIPTION

**Figure 1:**
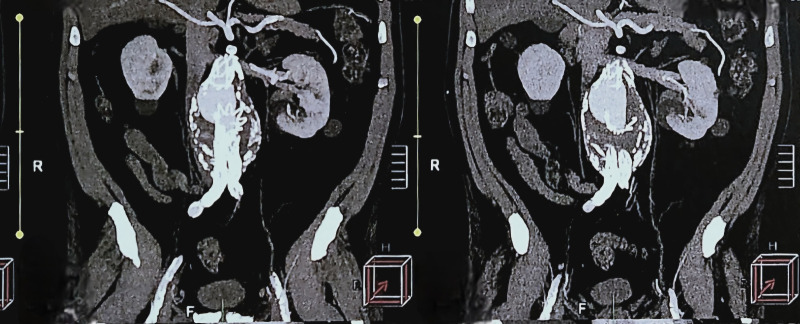
Showing the type IIIa endoleak in coronal computed tomography images, without contrast, in 2016.

A 65-year-old man who had undergone EVAR for an AAA in June 2005 reported that he had not had routine follow-up. He underwent computed tomography angiography, which found a large AAA, of around 7 cm, and a type IIIa endoleak, with the main endograft body separated from the juxtarenal proximal extension (Figure 1).

The report on the prior surgery from 2005 described placement of a 25x14x145 mm bifurcated Apolo endograft. The intraoperative arteriography indicated that the endograft free-flow had been positioned below the renal arteries. At the time, a 25x25x50 mm proximal extension had been used to attach the endograft, maintaining the free-flow at the renal arteries, with no signs of endoleak on the subsequent arteriography.

Initially, the patient had been asymptomatic and hemodynamically stable, taking valsartan, amlodipine, atorvastatin, and metformin. A reintervention was scheduled to fit two endografts to connect the main body of the original endograft to its proximal juxtarenal extension. This management approach was chosen because the type IIIa endoleak had probably occurred because of a failure of shape compatibility affecting the original deployment, rather than because of degradation of the endograft material.

For technical reasons – the original bilateral femoral approach and patient obesity – we employed ultrasound guided access to the femoral arteries and inserted a Medtronic Sentrant introducer sheath on the left.

**Figure 2:**
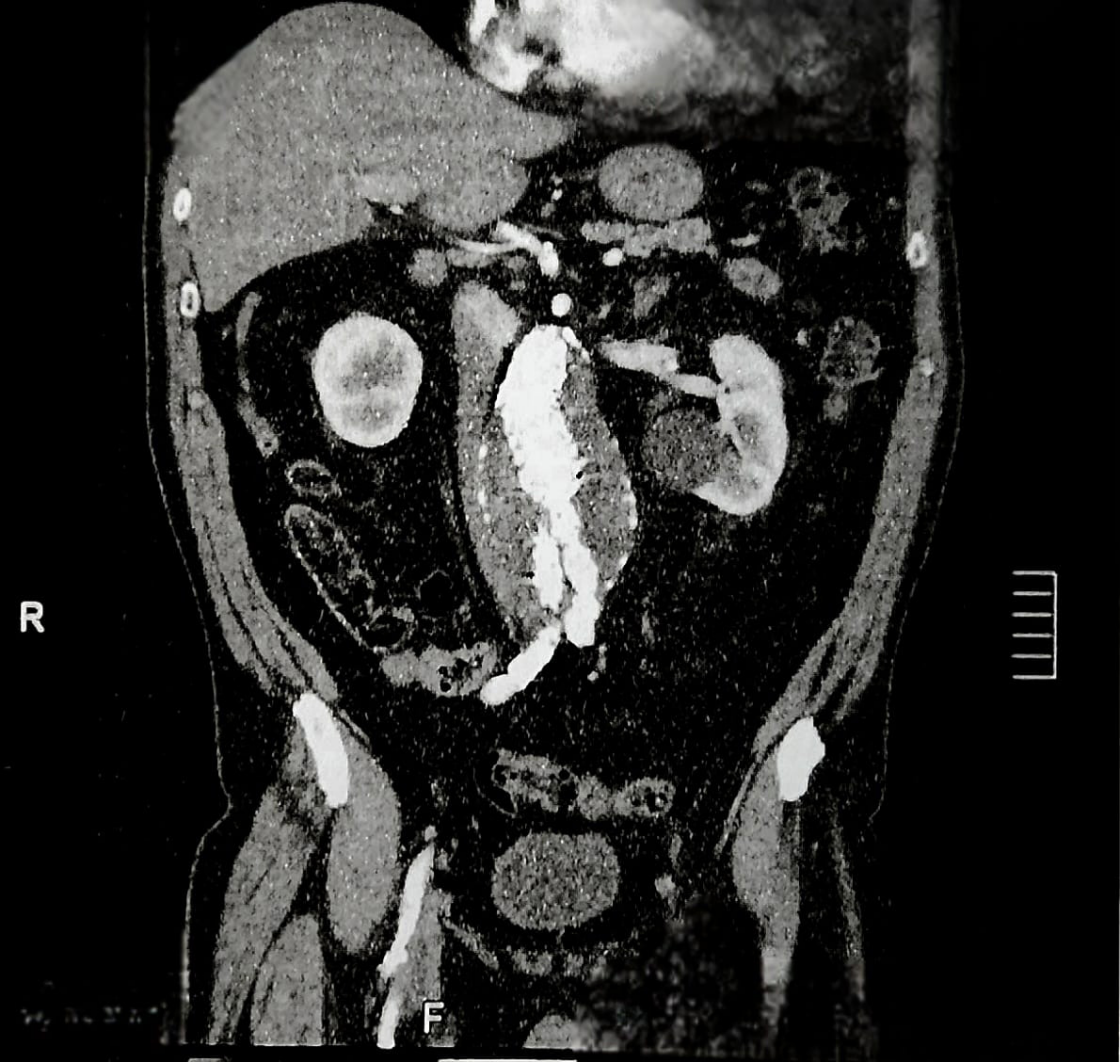
Postoperative computed tomography with contrast showing repair of the endoleak and maintenance of blood flow in the abdominal aorta, in 2016.

**Figure 3:**
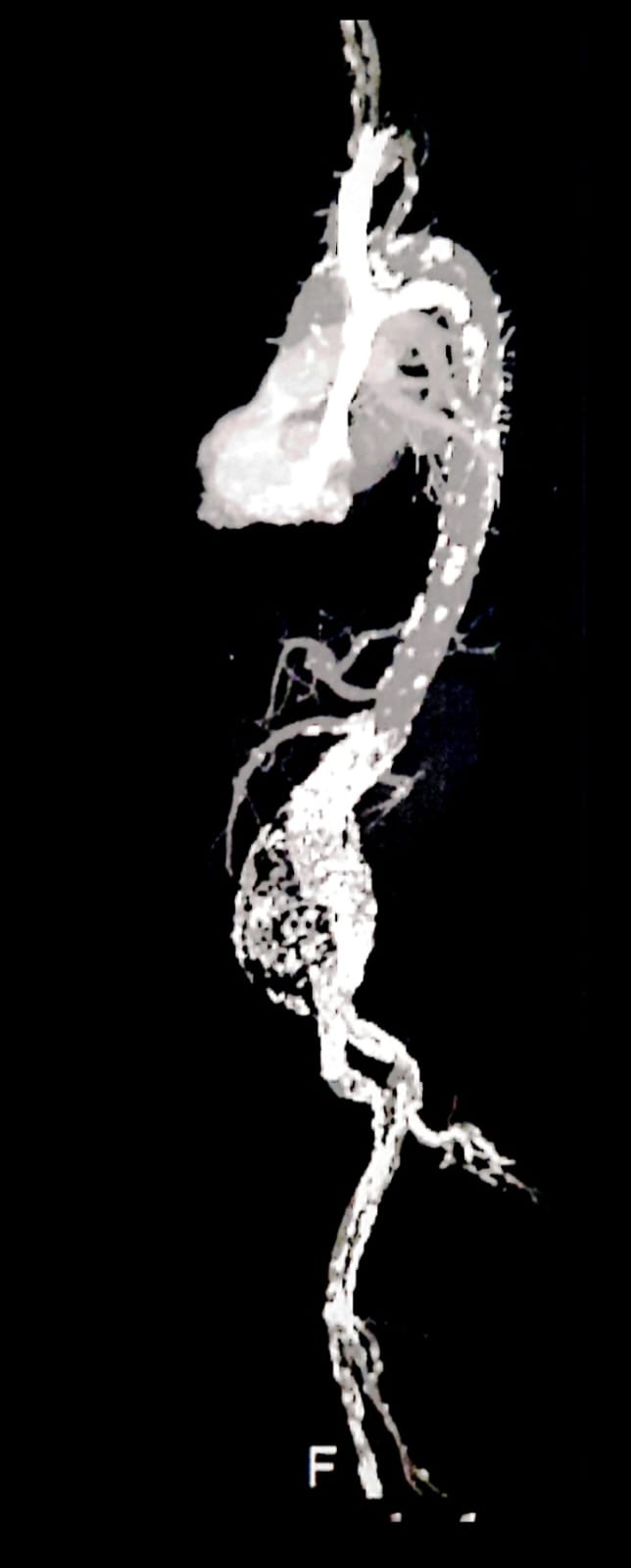
Control computed tomography angiography without contrast, showing the endograft structure intact and well positioned, in 2023.

**Figure 4:**
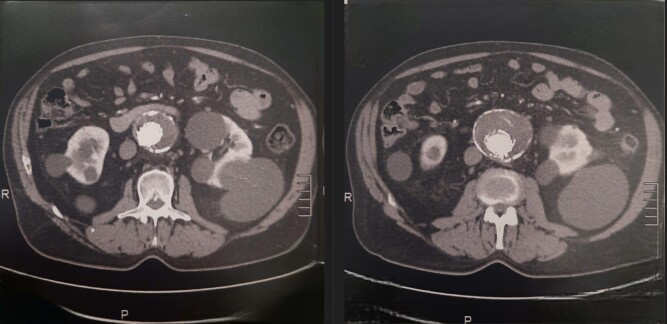
Computed tomography with contrast showing absence of blood flow into the aneurysm sac, in 2023.

The main body had a diameter of 25 mm and so two 28x28x70 mm Medtronic endografts were deployed from the renal arteries to the bifurcation of the original endograft. Ballooning was used to fit the endografts, followed by control angiography, showing excellent results and no presence of endoleaks. The femoral accesses were closed with ProGlide devices. The patient was discharged after 3 days in hospital. Figures 2, 3 and 4 show immediate postoperative imaging and follow-up 7 years after surgery.

## DISCUSSION

Abdominal aortic aneurysms account for more than 90% of aortic aneurysms, with prevalence ranging from 4 to 8% of the population, increasing progressively as age increases and occurring predominantly in men (at a proportion of 4:1).^1-6^ Type III endoleaks are caused by structural endograft failure, disintegration, or rupture of the material, enabling arterial blood flow with aneurysm expansion. More than half of these endoleaks are type IIIa.^1^

Surgery is indicated for AAA when diameter reaches or exceeds 5.5 cm in asymptomatic men and 5 cm in asymptomatic women, or in symptomatic patients with abdominal pains, signs of rupture, saccular aneurysms, or distal embolization.^1,4,6-8^ EVAR is preferred to open surgery because of the lower morbidity and mortality rates over both the short and long term, in addition to reduced rates of postoperative complications, although long term reintervention rates are higher.^1,5^

The endografts used in 2005 were Apolo brand, made of nitinol and polytetrafluoroethylene, with a 20 Fr profile and suprarenal anchoring. It is difficult to speculate in this report about the cause of the type IIIa endoleak. We can postulate certain hypotheses, such as: positioning of the free-flow of the first endograft below the renal arteries, the lack of greater oversizing of the proximal cuff, or aortic tortuosity, in addition to changes to endograft shape after deployment.^8^

There are postoperative control protocols for EVAR: radiological assessment with computed tomography and duplex scan of the abdominal aorta at 1, 6, and 12 months after repair to screen for enlargement of the aneurysm sac caused by possible endoleaks. In the absence of findings, annual screening is with duplex scan thereafter.^1,4,9^ Doppler ultrasonography with microbubble contrast (CEUS, contrast-enhanced ultrasound) is another diagnostic method that is being used and studied as an alternative to angiotomography for follow-up of these patients. This method can monitor blood flow through the endograft in real time without ionizing radiation or nephrotoxicity (contrast is provided by microbubbles which are then eliminated by the lungs). Studies suggest that CEUS offers superior sensitivity to serial computed tomography angiographies for identification of endoleaks in post-EVAR follow-up. Some protocols recommend CEUS as a supplementary method in cases of allergy to iodinated contrast or renal failure.^1,10,11^

According to the EVAR1^12^ and open vs. endovascular repair (OVER)^13^ studies and to the EUROSTAR^14^ protocol, 2.1% of endoleak*s* are type III,^15^ with a mean follow-up time at discovery of 5 to 6 years, varying from 1 to 13 years after EVAR.^5,16^ The endoleak in the patient in this case was identified in 2016, 11 years after the first intervention, confirming the literature, which reports a 60% failure rate after the first surgical intervention.^1^

The literature is categorical with regard to the relationship between the incidence of endoleaks and their causes after EVAR performed with first or second generation endografts and after EVAR with third generation devices. The earlier generations were used in procedures up to 1998 and have type III endoleak incidence rates from 8 to 12%, because of the smaller overlap recommended for these stent-grafts and delayed comprehension, on the part of surgeons, of the importance of fixation to the adjacent tissues. The incidence with third generation devices is just 1%, although the post-EVAR follow-up time is shorter.^5,17^

A retrospective study published in 2021 by Blakeslee-Carter et al.^15^ reported that 167 of a total of 4,070 patients who underwent EVAR for AAA had type III endoleaks (4.1%). Of these, 85% (133 cases) were type IIIa and almost 20% of the type III cases occurred in conjunction with another type, which was not observed in our patient. Moreover, just 0.7% of these patients needed reintervention after the endoleak was corrected by EVAR, over a 21-month follow-up period, which is considerably shorter than the 7 years in the case described here. That study found no relationship between presence of type III endoleak at hospital admission, rate of surgical intervention and mortality in these patients over 2 years’ follow-up. However, it was observed that the greater the modularity of the endografts used and the greater the modification performed by the surgeon before insertion, the greater the chance of development of type III endoleak.^15^ Both interventions in our patient were traditional.

EVAR is the procedure of choice for type III endoleak repair, with access and deployment of the new endograft inside of the original, covering the site of separation and eliminating blood flow into the aneurysm sac.^1-8^

In the case of our patient, positioning was difficult because of aneurysmal tortuosity and because of the distance between the separated parts of the original endograft.^5^ Since access to the suprarenal aorta was successful, there was no need to catheterize the brachial artery. Repair can be achieved with a tubular or bifurcated endograft, depending on the level of the separation or rupture. Thus, prior anatomic study of this patient’s aorta using computed tomography angiography was essential, enabling visualization of the level of separation and the distance between the parts, in addition to enabling the correct choice of two tubular endografts, connecting the main endograft body prior to the proximal juxtarenal extension. Use of a bifurcated endograft was ruled out because the endoleak was not caused by an intrinsic defect of the original endograft.

If the patient exhibits symptoms indicative of significant expansion of the aneurysm, imminent rupture or aortoduodenal or aortocaval fistula, open surgery or conversion should be chosen.^1,7,8,15^ While EVAR is safe, complications such as acute limb ischemia, mesenteric ischemia, or retroperitoneal bleeding can occur because of the procedure.^16,18,19^

Treatment is followed by the imaging exams recommended post-EVAR (computed tomography, duplex scan and, more recently, CEUS).^1,3,4,10,11^ Since this is a problem primarily related to the structure of the endograft, there is the possibility of another type III endoleak occurring in approximately 25%^12,16^ of cases, in addition to an up to nine times greater risk of aortic rupture, reinforcing the importance of post-intervention follow-up.

This case, therefore, highlights that endoleaks, the most common complication after EVAR for AAA, are overwhelmingly identified by control examinations during patient follow-up. While rare, type III endoleaks can be entirely and safely corrected using endovascular techniques to insert a second endograft to cover the failure in the first, providing the surgeon has the necessary experience and the correct materials to perform the procedure. This repair by EVAR proved functional and free from additional endoleak*s* after 7 years, highlighting the correctness of the therapeutic technique. Additional studies are needed to assess new follow-up methods, such as CEUS, which is a less invasive and more promising method. Emergence of type III endoleaks tends to diminish with the development of new stent-grafts, with more resistant and compliant materials, and it falls to the surgeon to assess the need to alter the shape of the device before insertion, considering the increased risk of postoperative complications.
