# Supplementary material for: Treatment of type IIIa endoleak and 7-year follow-up: case report
Source: J Vasc Bras. 2025 Jul 28;24:e20240019. doi: 10.1590/1677-5449.202400192 (PMC12419746; doi:10.1590/1677-5449.202400192)
Supplement: Translation

# Translation: Tratamento de *endoleak* tipo IIIa com seguimento de 7 anos: relato de caso

**DOI:** 10.1590/1677-5449.202400191

**Authors:** Luiz Ronaldo Godinho Pereira, Keller Soares Ávila, Vinicius Oliveira Godoi, Leonardo Augusto D’Avila Gonçalves, Rafael Fortes, Marcelo Adriano de Assis Hudson, Daniel Mendes Pinto

**Affiliations:** 1 Hospital Márcio Cunha, Ipatinga, MG, Brasil.; 2 Universidade Federal de Juiz de Fora – UFJF, Juiz de Fora, MG, Brasil.; 3 Hospital Felício Rocho, Belo Horizonte, MG, Brasil.

**Keywords:** endoleak, aneurisma aórtico, diagnóstico por imagem

## Abstract

O *endoleak* tipo III é caracterizado por um problema estrutural da endoprótese, como fratura da estrutura metálica, desconexão ou ruptura tecidual. Trata-se de uma complicação rara, ocorrendo em 2,1% dos pacientes após tratamento de aneurisma de aorta abdominal por reparo endovascular, com incidência maior em primeira e segunda gerações de endopróteses, podendo ocorrer precoce (após 30 dias) ou tardiamente. É dividido em IIIa – separação modular do componente – e IIIb – fratura da malha ou ruptura envolvendo a endoprótese. Este é um relato de caso de um paciente assintomático, previamente submetido à correção de aneurisma de aorta abdominal infrarrenal, que realizou uma tomografia computadorizada de seguimento, sendo encontrado um *endoleak* tipo IIIa com desconexão do corpo principal à extensão proximal. Uma nova abordagem endovascular terapêutica foi realizada para selamento do *endoleak* e correção aneurismática.

## INTRODUÇÃO

O reparo endovascular (EVAR, do termo em inglês *endovascular aneurysm repair*) é a técnica mais utilizada para o tratamento do aneurisma de aorta abdominal (AAA). Entretanto, algumas complicações podem ocorrer, sendo a principal o *endoleak*, que é a persistência de fluxo sanguíneo para o saco aneurismático após EVAR, representando cerca de 33% das complicações, embora cerca de 50% dos casos se resolvam espontaneamente^1-4^.

Os *endoleaks* são divididos em cinco tipos: I, separado em proximal (Ia) e distal (Ib); II, quando há crescimento do saco aneurismático por fluxo sanguíneo retrógrado de vasos; III, quando há desconexão da endoprótese, seja no corpo ou no membro contralateral (IIIa), rasgo ou fratura na endoprótese (IIIb); IV, decorrente da porosidade da endoprótese; e V, quando há aumento aneurismático na ausência de *endoleak* (endotensão)^1-4^.

O tratamento do tipo III é obrigatório, visto que há pressurização direta do aneurisma e alto risco de ruptura^1,5^. Este trabalho relata um caso no qual se utilizou a técnica totalmente endovascular para correção desse tipo de *endoleak*.

Este relato de caso foi aprovado pelo Comitê de Ética e Pesquisa do Hospital Felício Rocho, Belo Horizonte, Brasil, com Certificado de Apresentação de Apreciação Ética 79209524.4.0000.5125. Número do Parecer Consubstanciado: 6.801.488.

## DESCRIÇÃO DO CASO

Homem de 65 anos relatou ter sido submetido à correção de AAA por EVAR em junho de 2005, sem realização de controle de rotina. Foi submetido, então, à angiotomografia computadorizada, que identificou AAA volumoso, com cerca de 7 cm, associado a *endoleak* tipo IIIa, mostrando desconexão do corpo principal da endoprótese à extensão proximal justarrenal (Figura 1).

**Figura 1:** Visualização de *endoleak* tipo IIIa em tomografia computadorizada, sem contraste, em corte coronal, em 2016.

A descrição prévia da cirurgia de 2005 indicava a colocação de uma endoprótese Apolo bifurcada de 25x14x145 mm. A arteriografia intraoperatória indicou descida da endoprótese e o posicionamento do *free-flow* abaixo das artérias renais. À época, optou-se pela passagem de uma extensão proximal de 25x25x50 mm para fixação da endoprótese, mantendo o *free-flow* sobre as artérias renais, sem sinais de *endoleak* na nova arteriografia.

Inicialmente, o paciente encontrava-se assintomático e hemodinamicamente estável, em uso de valsartana, anlodipino, atorvastatina e metformina. Optou-se por nova abordagem, com a colocação de duas endopróteses conectando o corpo principal da endoprótese já existente à extensão proximal justarrenal. Essa abordagem foi escolhida devido ao *endoleak* tipo IIIa provavelmente ter ocorrido por falta da conformidade na implantação prévia, e não por degradação do tecido da endoprótese.

Por questões técnicas – abordagem prévia das femorais bilateralmente e obesidade –, fizemos acesso nas artérias femorais por punções guiadas por ultrassom, e implante do transdutor Sentrant, da Medtronic, à esquerda.

O corpo principal tinha diâmetro de 25 mm. Optou-se, portanto, pela colocação de duas endopróteses Medtronic de 28x28x70 mm, estendendo-se das artérias renais à bifurcação da endoprótese previamente implantada. Foi realizado balonamento para acomodação das endopróteses e controle angiográfico, com ótimo resultado e sem presença de *endoleak*. O fechamento das femorais foi feito com o dispositivo ProGlide. O paciente recebeu alta após 3 dias internado. As Figuras 2, 3 e 4 mostram o acompanhamento por imagem imediatamente após o procedimento e 7 anos após a cirurgia.

**Figura 2:** Tomografia computadorizada contrastada do pós-operatório evidenciando reparo do *endoleak* e manutenção do fluxo sanguíneo da aorta abdominal, em 2016.

**Figura 3:** Angiotomografia computadorizada sem contraste, de controle, evidenciando a estrutura da endoprótese sem desabamento e bem localizada, em 2023.

**Figura 4:** Tomografia computadorizada contrastada evidenciando ausência de fluxo sanguíneo para saco aneurismático, em 2023.

## DISCUSSÃO

O AAA representa mais de 90% dos aneurismas aórticos, com prevalência de 4 a 8% na população e aumento progressivo conforme a idade, ocorrendo mais em homens (proporção de 4:1)^1-6^. O *endoleak* tipo III resulta de falha estrutural da endoprótese, desintegração ou dilaceração do material, permitindo fluxo sanguíneo arterial com crescimento do aneurisma. Entre as ocorrências desse tipo, mais da metade são do tipo IIIa^1^.

A cirurgia do AAA é indicada quando o diâmetro atinge 5,5 cm ou mais em homens assintomáticos, 5 cm em mulheres assintomáticas, ou em pacientes sintomáticos com dor abdominal, sinais de ruptura, aneurismas do tipo sacular e embolização distal^1,4,6-8^. Prefere-se o EVAR em relação à cirurgia aberta devido às menores taxas de morbimortalidade a curto e longo prazo, além da redução de complicações pós-operatórias, embora a taxa de reintervenção a longo prazo seja maior^1,5^.

As endopróteses utilizadas em 2005 foram a Apolo, constituída por nitinol e politetrafluoretileno, com perfil de 20 Fr e fixação suprarrenal. É difícil opinar, neste relato, sobre a causa do *endoleak* tipo IIIa. Podemos idealizar algumas hipóteses, como: o posicionamento do *free-flow* da primeira endoprótese abaixo da renais, a falta de um maior *oversize* do *cuff* proximal ou a própria tortuosidade aórtica, além da alteração da conformação após colocação da endoprótese^8^.

Existem protocolos para controle pós-operatório: avaliação radiológica com tomografia computadorizada e *duplex scan* da aorta abdominal após 1, 6 e 12 meses do reparo para rastreio de alargamento do saco aneurismático por possível *endoleak*. Sem isso, prossegue-se com rastreio anual com *duplex scan* ^1,4,9^. O eco-Doppler associado ao contraste de microbolhas (CEUS, do termo em inglês *contrast-enhanced ultrasound*) é outro método diagnóstico que vem sendo utilizado e estudado como alternativa à angiotomografia no acompanhamento desses pacientes. Esse método permite monitorar em tempo real o fluxo sanguíneo pela endoprótese, sem radiação ionizante ou nefrotoxicidade (o contraste é feito por microbolhas que são eliminadas pelo pulmão). Estudos sugerem que o CEUS apresenta sensibilidade superior a angiotomografias seriadas para identificação de *endoleaks* no seguimento pós-EVAR. Alguns protocolos recomendam o CEUS como método complementar nos casos de alergia ao contraste iodado ou insuficiência renal^1,10,11^.

Conforme estudos EVAR 1^12^ e *open versus endovascular repair* (OVER)^13^, e o protocolo EUROSTAR^14^, 2,1% dos *endoleaks* enquadram-se no tipo III^15^, sendo a média de acompanhamento para descoberta de 5 a 6 anos, variando de 1 a 13 anos após o EVAR^5,16^. O *endoleak* no paciente em questão foi identificado em 2016, 11 anos após a primeira abordagem, corroborando a literatura, que alega uma taxa de 60% de falhas no seguimento dos pacientes após a primeira intervenção cirúrgica^1^.

A literatura é categórica quanto à relação entre o EVAR com endopróteses de primeira ou segunda geração e aquelas de terceira geração, no que se refere à incidência de *endoleak* e suas causas. Os primeiros, utilizados em procedimentos até 1998, apresentam incidência de 8 a 12% de *endoleak* tipo III, devido à pequena sobreposição recomendada para esses *stents* e à compreensão tardia, por parte dos cirurgiões, da importância da fixação ao tecido adjacente. Já na terceira geração, a incidência é de apenas 1%, embora o tempo de acompanhamento pós-EVAR seja menor^5,17^.

Blakeslee-Carter et al.^15^, em estudo retrospectivo de 2021, descreveram que, de 4.070 pacientes que realizaram EVAR para tratamento de AAA, 167 apresentaram *endoleak* tipo III (4,1%). Desses, 85% (133 casos) eram do tipo IIIa, e quase 20% dos casos de tipo III ocorreram concomitantemente com outro tipo, algo não observado em nosso paciente. Ademais, apenas 0,7% dos pacientes relatados necessitaram de nova intervenção após correção do *endoleak* por EVAR, em acompanhamento de 21 meses, período consideravelmente inferior ao de 7 anos do paciente relatado. Tal estudo não evidenciou relação entre a presença de *endoleak* tipo III na admissão hospitalar, taxa de intervenção cirúrgica e mortalidade desses pacientes em um seguimento de 2 anos. Contudo, observou-se que, quanto maior a modularidade das endopróteses usadas e quanto mais modificada a endoprótese pelo cirurgião antes da inserção, maior a chance de desenvolvimento de *endoleak* tipo III^15^. Em nosso paciente, ambas as abordagens foram tradicionais.

O EVAR é o procedimento de escolha para reparo de *endoleak* tipo III, com acesso e posicionamento de nova endoprótese internamente à antiga, cobrindo o local da desconexão e eliminando fluxo sanguíneo para o saco aneurismático^1-8^.

O posicionamento, no caso do nosso paciente, foi dificultado pela tortuosidade aneurismática e distância entre as partes desconexas da endoprótese^5^. Como o acesso na aorta suprarrenal foi bem-sucedido, não houve necessidade de cateterização da artéria braquial. Para a correção, a nova endoprótese pode ser tubular ou bifurcada, dependendo do nível da desconexão ou ruptura. Logo, o estudo anatômico prévio da aorta desse paciente por meio da angiotomografia computadorizada foi essencial, permitindo a visualização do nível da desconexão e da distância entre as partes, além de possibilitar a indicação correta de duas endopróteses tubulares, conectando o corpo principal da endoprótese prévia à extensão proximal justarrenal. O implante de endoprótese bifurcada foi descartado, devido ao fato de o *endoleak* não ter se originado de um defeito intrínseco da endoprótese.

Caso o paciente apresente sintomas que indiquem alargamento importante aneurismático, eminente ruptura ou fístula aortoduodenal ou aortocaval, opta-se pela cirurgia aberta ou conversão^1,7,8,15^. Embora o EVAR seja seguro, complicações como isquemia aguda de membro, isquemia mesentérica ou sangramento retroperitoneal podem ocorrer devido ao procedimento^16,18,19^.

O tratamento é seguido por exames de imagem preconizados após EVAR (tomografia computadorizada, *duplex scan* e, mais recentemente, CEUS)^1,3,4,10,11^. Por ser um problema relacionado principalmente à estrutura da endoprótese, há a possibilidade de ocorrência de um novo *endoleak* tipo III em aproximadamente 25%^12,16^ dos casos, além de um aumento de até nove vezes no risco de ruptura aórtica, corroborando a importância do seguimento pós-abordagem.

Esse caso, portanto, reforça que o *endoleak*, principal complicação pós-EVAR para correção de AAA, é identificado, sobretudo, por exames de controle no seguimento do paciente. O tipo III, embora raro, pode ser totalmente corrigido com técnicas endovasculares de forma segura, mediante a inserção de uma segunda endoprótese que recubra a falha da primeira, desde que o cirurgião tenha experiência e os materiais corretos para realizar o procedimento. Esse reparo por EVAR se mostrou funcional e sem novos *endoleaks* após 7 anos, ressaltando a assertividade da técnica terapêutica. Novos estudos devem ser realizados para avaliar novas formas de rastreio, como o CEUS, que se trata de um método menos invasivo e mais promissor. O surgimento de *endoleaks* tipo III tende a reduzir com o desenvolvimento de novos *stents*, com materiais mais resistentes e complacentes, e cabe ao cirurgião avaliar a necessidade de alterar a conformação da peça antes da inserção, considerando o maior risco de complicação pós-operatória.
